# Mid upper arm circumference as a predictor of risk of mortality in children in a low resource setting in India

**DOI:** 10.1371/journal.pone.0197832

**Published:** 2018-06-01

**Authors:** Sunita Taneja, Temsunaro Rongsen-Chandola, Sanjana Brahmawar Mohan, Sarmila Mazumder, Nita Bhandari, Jasmine Kaur, Nikita Arya, Ranadip Chowdhury, Jose Carlos Martines, Rajiv Bahl, M. K. Bhan

**Affiliations:** 1 Centre for Health Research and Development, Society for Applied Studies, New Delhi, India; 2 Centre for Intervention Science in Maternal and Child Health, Centre for International Health, University of Bergen, Norway; 3 Department of Maternal, Newborn, Child and Adolescent Health, World Health Organisation, Geneva, Switzerland; 4 Indian Institute of Technology, New Delhi, India; Institut de recherche pour le developpement, FRANCE

## Abstract

**Objective:**

In this secondary analysis of data from an intervention trial, we assessed the performance of Mid Upper Arm Circumference (MUAC) as a predictor of mortality in children aged 6–59 months from Delhi, India, one year after their initial MUAC measurements were taken. Additionally, we assessed MUAC as an absolute value and MUAC z-scores as predictors of risk of mortality.

**Methods:**

In the trial, children were screened using MUAC prior to referral to the study clinic. These children were revisited a year later to ascertain their vital status. Baseline MUAC and MUAC z-scores were used to categorize children as severely (MUAC <115 mm, MUAC z-score <-3SD) or moderately (MUAC 115 to <125 mm, MUAC z-score <-2SD) malnourished. The proportion of malnutrition, risk of mortality, relative risk estimates, positive predictive value and area under the curve (AUC) by MUAC and MUAC z-scores were calculated.

**Results:**

In the resurvey, the first 36159 children of the 48635 in the initial survey were contacted. Of these, vital status of 34060 (94.2%) was available. The proportion of severe malnutrition by MUAC (<115 mm) was 0.5% with an associated mortality of 4.7% over a one year period and an attributable mortality of 13% while the proportion of the severe malnutrition by MUAC z-score (<-3SDwas 0.9% with an associated mortality of 2.2%.

**Conclusions:**

MUAC is a significant predictor of subsequent mortality in under-five children. In settings where height measurement is not feasible, MUAC can be used as a screening tool for identifying severely malnourished children for management.

## Introduction

Severe acute malnutrition (SAM) in children aged 6–59 months is defined either by weight-for-height z- score (WHZ) <-3 SD or Mid Upper Arm Circumference (MUAC) < 115 mm or presence of edema feet [1, 2, 3].

The current WHO recommendation is to use MUAC as a screening tool in the community while at the health facility level to use MUAC or WHZ or bilateral oedema as independent admission criteria for management of SAM [3]. MUAC as a screening tool has been demonstrated to be a better predictor of mortality in several countries [4–8] which may be explained by the fact that MUAC selects younger children as malnourished compared to WHZ [9].

Countries interested in addressing SAM in under-five children might prefer the use of MUAC because of its operational ease, though there is often uncertainty among program managers about its appropriateness relative to WHZ. It is known that WHZ and MUAC identify different sets of children with some overlap, and the level of agreement between the two methods has varied across studies by approximately 50–60% [1, 10–16]. However, both MUAC and WHZ are reported to have prognostic ability to predict mortality in children [17–21].

Program managers are also concerned whether the ability of MUAC to predict mortality varies in different regions of the world given variations in the clinical presentation of SAM [22, 23]. There is a view that SAM in India is a chronic or acute on chronic condition.

An examination of how well MUAC predicts mortality in under-five children is important in settings like India. This could provide invaluable information for program managers who are considering using MUAC to identify SAM children in the community for home based treatment programs.

We examined both MUAC and MUAC z-scores as predictors of risk of mortality.

We report findings from a secondary analysis of data from an intervention trial comparing options for home treatment of SAM where the initial screening of children was by MUAC [24]. A revisit was made a year later in children in whom MUAC was measured, to ascertain vital status. These findings will be of value as efforts are being made to scale up programs for treating SAM children in India.

## Material and methods

This analysis uses data from a multicentre intervention trial; however the data used for this analysis is from the Delhi site. The detailed methods used have been published previously [24]. Briefly, the study was done in low resource urban areas in Delhi. The study population was multi-ethnic. The median years of schooling among fathers was 7.1 (IQR 4.3) and of mothers it was 4.7 (IQR 4.6). In this population, access to piped water supply was limited (18%) and around half (49%) the families had toilets inside the house. Children aged 6–59 months were identified in a household survey and MUAC was measured. Those with MUAC <130 mm were then assessed by WHZ. Those with WHZ <-3 SD or edema of the feet and without severe illness requiring hospitalization were enrolled into the intervention trial. We aimed to visit all children measured in the survey a year later to ascertain information on vital status. However, as the study was ending we could only visit the first 36159 children.

MUAC was measured using a non stretch tape (Chasmors CTM03; accuracy 1 mm) by trained study workers who had all undergone inter and intra observation standardization exercises.

Ascertainment of vital status during the revisit was done by an independent team. This team ensured that all households were revisited in the same order that they were surveyed during the initial household survey. Special care was taken to correctly identify a child by using multiple variables like name of head of household, name of child, father’s name, and mother’s name. In non available families at least three home visits were made over two weeks.

### Ethical approval

The main intervention trial was approved by the institutional Ethics Committee of each participating institution. The Delhi site approval was given by the Society for Applied Studies Ethics Review Committee (SAS ERC/40/2012). Written informed consent was obtained from parents of all children. Permission was obtained from the Principal Investigator for the use of the data for secondary analysis.

### Statistics

Data were analyzed using Stata software version 13.1 (Stata Corporation, College Station, TX). MUAC z- scores were calculated using the WHO Anthro software [25]. We defined MUAC <115 mm as severe malnutrition and MUAC 115 to <125 mm as moderate malnutrition [3]. Likewise, MUAC z-score <-3 SD was defined as severe malnutrition and MUAC z-score <-2 SD as moderate malnutrition [1]. Only the deaths that occurred within 12 months of the MUAC measurement were included in the analysis. The proportion of children with severe and moderate malnutrition and attributable morality proportion was estimated for both MUAC and MUAC z-scores. Multinomial logistic regression was used to estimate relative risk (RR) of mortality among severe and moderate malnourished children defined by both MUAC and MUAC z-score.

We created a combined indicator using MUAC and MUAC z score; MUAC<115 mm and MUAC z <-3 SD, MUAC <115 mm and MUAC z ≥-3 SD, MUAC ≥115 mm and MUAC-z <-3 SD, MUAC ≥125 mm and MUAC z ≥ -2 SD. Multinomial logistic regression was used to estimate relative risk (RR) of mortality.

Sensitivity of MUAC z-score and MUAC was defined as the proportion of children who died among those who were below the cut-off. Specificity was defined as the proportion of surviving children among those who were above the cut-off. The positive predictive value (PPV) was defined as the proportion of children who died among children classified as malnourished. The negative predictive value (NPV) was defined as the proportion of children who did not die among those not classified as malnourished. For a given marker, area under the curve (AUC) is an index of the marker’s ability to discriminate between true positives and true negatives. AUC was calculated to evaluate the overall prognostic ability of MUAC and MUAC z-score to detect mortality. For the specific MUAC or MUAC-z cut-offs, we used the ‘diagti’ command in Stata to calculate AUC. We also plotted ROC curves and area under curves with 95% CI for the overall continuous MUAC- z and MUAC data.

## Results

**Fig 1:**
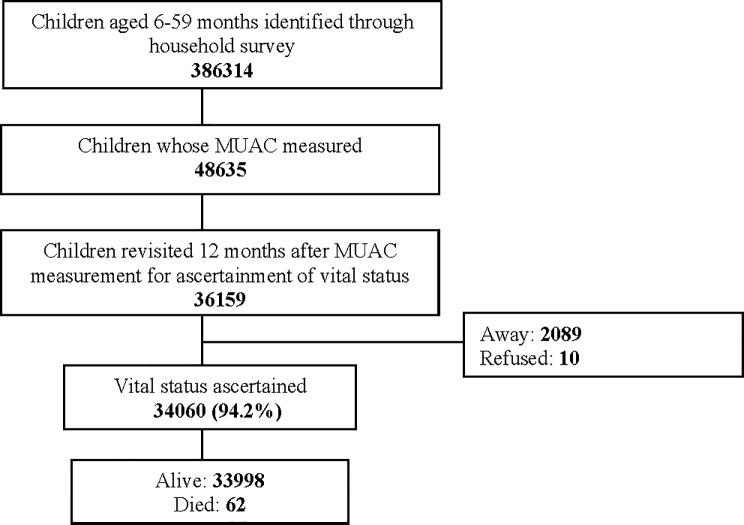
Trial profile.

In the time available prior to the end of the funding period of the intervention trial, we were able to visit the first 36159 children of the 48635 measured in the initial survey. Information on vital status could be obtained in 34060 (94.2%) children (Fig 1). Of these, 34002 (99.8%) were alive and 62 (0.2%) had died. Of the remaining 2099 (5.8%) children, 2089 families could not be contacted despite 3 visits over a period of two weeks to the household and 10 (0.5%) families refused (Fig 1). Around half (47.7%) of the children were female (data not shown), 34% were aged 6–23 months and 66% were 24–59 months old (Table 1).

**Table 1:** Mortality, attributable mortality and risk of mortality in children aged 6–59 months over a 12 month period following initial MUAC measurement by MUAC category.

**Overall: Children aged 6–59 months (total number of children 34060)**				
MUAC (mm)	Proportion	Mortality	Attributable mortality %	RR (95% CI)
<115	169 (0.5)	8 (4.7)	13	37.64 (17.4, 81.3)
≥115-<125	1274 (3.7)	11 (0.9)	18	6.59 (3.4, 12.8)
≥125	32617 (95.8)	43 (0.1)	69	Reference
**Children aged 6–23 months (Total number of children 11466)**				
<115	140 (1.22)	6 (4.3)	17	24.29 (9.5, 61.8)
≥115-<125	999 (8.7)	10 (1)	28	5.48 (2.5, 11.8)
≥125	10327 (90.0)	19 (0.2)	54	Reference
**Children aged 24–59 months (Total number of children 22594)**				
<115	29 (0.1)	2 (6.9)	7	68.72 (15.5, 305.2)
≥115-<125	275 (1.2)	1 (0.4)	4	3.38 (0.4, 25.1)
≥125	22290 (98.6)	24 (0.1)	89	Reference

Figures are number (percentage) unless stated otherwise

In children aged 6–59 months, the proportion with MUAC <115mm was 0.5% with an associated mortality of 4.7%, an attributable mortality of 13% and a risk of mortality of 37.64 (17.4, 81.3) compared to children with MUAC ≥125 mm. The proportion with MUAC between ≥115 and <125 mm was 3.7% with a mortality of 0.9%, an attributable mortality of 18% and a risk of mortality of 6.59 (95% CI: 3.4 12.8) compared to children with MUAC ≥125 mm. The risk of mortality in children with MUAC <115 mm was 24.29 (95% CI: 9.5, 61.8) in those aged 6–23 months while it was 68.72 (15.5, 305.2) in those aged 24–59 months compared to children with MUAC ≥125 mm.

The prevalence of MUAC <115 mm, between ≥115 to <125 mm and ≥ 125 mm in relation to mortality, attributable mortality and risk of mortality in the age groups 6–23 and 24–59 months is also shown in Table 1.

In children aged 6–59 months, the proportion with MUAC z-score <-3 was 0.9% with an associated mortality of 2.2%, an attributable mortality of 11% and a risk of mortality of 17.04 (95% CI: 7.6, 38.3) compared to children with MUAC for age z-score ≥-2. The proportion with MUAC z-score between ≥-3 and <-2 was 9.4% with a mortality of 0.5%, an attributable mortality of 24% and a risk of mortality of 3.58 (95% CI: 1.9, 6.5) compared to children with MUAC for age z score ≥-2. The risk of mortality in children with MUAC z-score <-3 was 16.78 (95% CI: 5.6, 49.8) in those aged 6–23 months and it was 15.95 (95% CI: 4.7, 54.1) in those aged 24–59 months compared to children with MUAC for age z score ≥-2 (Table 2).

**Table 2:** Mortality, attributable mortality and risk of mortality in children aged 6–59 months over a 12 month period following initial MUAC measurement by MUAC z-score.

**Overall: Children aged 6–59 months (Total number of children 34060)**				
MUAC z-score	Proportion	Mortality	Attributable^3^ mortality %	RR (95% CI)
<-3	320. (0.9)	7 (2.2)	11	17.04 (7.6, 38.3)
≥-3-<-2	3207 (9.4)	15 (0.5)	24	3.58 (1.9, 6.5)
≥-2	30533 (89.9)	40 (0.1)	65	Reference
**Children aged 6–23 months (Total number of children 11466)**				
<-3	129 (1.1)	4 (3.1)	11	16.78 (5.6, 49.8)
≥-3-<-2	824 (7.2)	11 (1.3)	31	7.09 (3.4, 14.8)
≥-2	10513 (91.7)	20 (0.2)	57	Reference
**Children aged 24–59 months (Total number of children 22594)**				
<-3	191 (0.8)	3 (1.6)	11	15.95 (4.7, 54.1)
≥-3-<-2	2383 (10.5)	4 (0.2)	15	1.68 (0.6, 4.9)
≥-2	20020 (88.6)	20 (0.1)	74	Reference

Figures are number (percentage) unless stated otherwise

The prevalence of MUAC for age z -score <-3SD, between ≥-3 to <-2 and ≥-2 in relation to mortality, attributable mortality and risk of mortality in the age groups 6–23 and 24–59 months are also shown in Table 2.

Of the 169 children with MUAC <115 mm, 59 had received one of the three feeding regimens of the intervention trial. The associated mortality in these 59 children was 1.6% (1/59) as against 6.3% (7/110) in those who did not participate in the trial. In children with a MUAC z score <-3SD, 113 participated in the intervention trial. The associated mortality in these 113 children was 1.76% (2/113) while it was 2.38% (5/210) in those that did not participate in the study (data not shown).

In children with a MUAC z-score <-3SD, the sensitivity of diagnosing probability of mortality by MUAC z-score was 11.3 (4.7, 21.9) and the specificity was 99.1 (99, 99.2). In children with MUAC <115 mm, the sensitivity was 12.9 (5.7, 23.9) and specificity was 99.5 (99.4, 99.6).

The area under the curve for MUAC z-score and MUAC was similar by cut-offs for severe and for combined severe and moderate malnutrition groups (Table 3).

**Table 3:** Diagnostic probability of mortality by MUAC z-scores and MUAC categories in children aged 6–59 months.

MUAC z-score		MUAC	
**Sensitivity/Specificity (95% CI)**			
<-3 SD	11.3 (4.7, 21.9) /99.1(99, 99.2)	<115 mm	12.9 (5.7, 23.9) / 99.5 (99.4, 99.6)
<-2 SD	35.5 (23.7, 48.9) /89.7 (89.9, 90)	<125 mm	30.6 (19.6, 43.7) /95.8 (95.6, 96.0)
**PPV**^**1**^**/NPV**^**2**^**(95% CI)**			
<-3 SD	2.2 (0.9, 4.5) /99.8 (99.8, 99.9)	<115 mm	4.7 (2.1, 9.1) / 99.8 (99.8, 99.9)
<-2 SD	0.6 (0.4, 0.9) /99.9 (99.8, 99.9)	<125 mm	1.3 (0.8, 2.0) / 99.9 (99.8, 99.9)
**AUC**^**3**^ **(95% CI)**			
<-3 SD	0.55 (0.51, 0.59)	<115 mm	0.56 (0.52, 0.60)
<-2 SD	0.63 (0.57, 0.69)	<125 mm	0.63 (0.57, 0.69)

^1^PPV: Positive Predictive Value

^2^NPV: Negative Predictive Value

^3^AUC: Area Under the Curve

The AUC analysis for MUAC <115 mm remained the same at 0.56 (95% CI; 0.52, 0.60) when the 323 children who were part of the intervention trial were excluded.

The diagnostic probability of mortality by MUAC z-score and MUAC categories in children of different age groups are given in the supplementary tables (S1 and S2 Tables).

The risk of mortality in children with MUAC <115 mm and MUAC z <-3SD was 40.67 (95% CI; 16.9, 98.0) compared to children with MUAC ≥125 mm and MUAC z ≥ -2 SD. While the risk of mortality in children with MUAC <115 mm and MUAC z ≥-3SD was 36.05, (95% CI 8.4, 154.0), and those with MUAC ≥115 mm and MUAC z <-3SD was 4.04 (95% CI 0.5, 29.6) compared to children with MUAC ≥125 mm and MUAC z ≥ -2 SD (Table 4).

**Table 4:** Mortality and risk of mortality in children aged 6–59 months of age by combined categories of MUAC and MUAC z-score.

Category	Proportion	Mortality	RR (95% CI)
MUAC<115 mm and MUAC z <-3 SD	123 (0.4)	6 (4.9)	40.67 (16.9, 98.0)
MUAC <115 mm and MUAC z ≥-3 SD	46 (0.1)	2 (4.3)	36.05 (8.4, 154.0)
MUAC ≥115 mm and MUAC z <-3 SD	197 (0.6)	1 (0.5)	4.04 (0.5, 29.6)
MUAC ≥125 mm and MUAC z ≥ -2 SD	30177 (99)	38 (0.12)	Reference

Figures are number (percentage) unless stated otherwise

**Fig 2:**
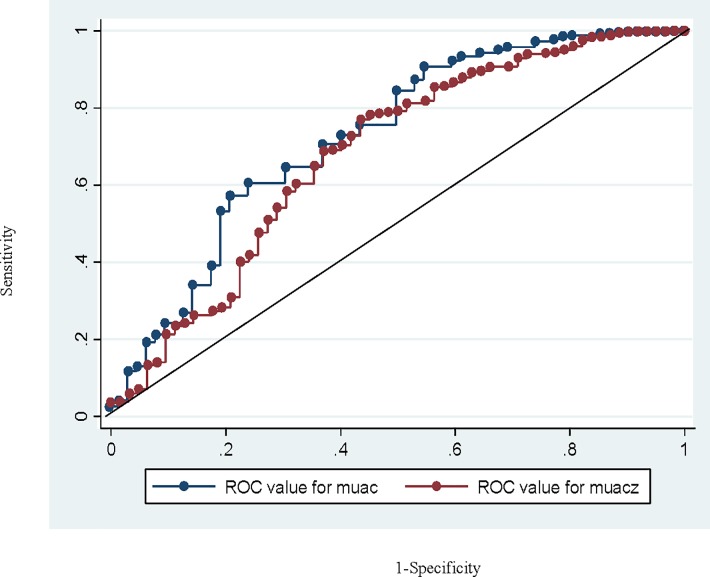
ROC curve of MUAC and MUAC z-score.

Fig 2 shows ROC curves and areas for the overall continuous MUAC-z and MUAC data for predicting mortality. The AUC (95%CI) for MUAC and MUAC z was 73.6 (65.9 to 80.3) and 67.9 (60.2 to 75.7) respectively.

## Discussion

In this setting, MUAC < 115mm and MUAC z-score <-3SD were significant predictors of mortality in under-five children. Further, MUAC and MUAC z-scores were similar in predicting mortality in under-five children comparable to findings from Guinea-Bissau [18]. These findings are consistent with the recommendation that MUAC may be used as a screening tool for identifying children with SAM in the community. The WHO recommends either MUAC <115 mm or WHZ z-scores <-3SD or bilateral pedal edema as suitable for selecting children with severe malnutrition for rehabilitation. The observed mortality rate in children with MUAC <115 mm is most likely an underestimate as some of these children had received rehabilitation as a result of participation in the intervention trial. Further a combination of MUAC <115 mm and MUAC z-score <-3SD gave a higher prediction of mortality risk compared to MUAC <115 mm but MUAC z-score ≥ -3SD.

Since MUAC <115 mm and WHZ <-3SD identify different subsets of at risk children, it is important to find a way to identify and select the largest proportion of children at significant mortality risk in programs for rehabilitation at facilities and at home. One limitation of the current analysis is that WHZ data were not available for all the children and therefore, this criteria could not be concurrently evaluated or compared for prediction of mortality.

Ideally, in a program one would like to identify as many as possible of the children who are at higher risk of subsequent mortality as a result of severe malnutrition. This would require an approach that judicially uses MUAC as a screening tool in households and community surveys followed by measurement of weight and height of the screened in children at a facility where it is feasible to obtain these measurements.

Others have suggested a two step process where children with MUAC <125 mm are identified as a first step followed by measurement of weight and height of the screened in children and selection of those with MUAC <115 or WHZ <-3SD for rehabilitation [26]. This strategy is supported by observations in Sierra Leone, where children with MUAC <125 mm cut-off showed a 83% recovery and 71% coverage while children identified by WHZ <-2 SD had a 79% recovery with 55% community coverage [27].

In any setting where measurement of height is simply not possible, then selection of children for rehabilitation using MUAC <115mm may be an acceptable option.

The primary strengths of the study are its very large size and true unselected population. The finding that MUAC z is no better than absolute MUAC in predicting survival echoes findings from Africa [13].

The limitation of this dataset is that information on the cause and timing of death in these children was not available. It is therefore, not possible to comment if severe malnutrition as defined by either MUAC or MUAC z led to the death of these children.

This dataset can only demonstrate that children with low MUAC or MUAC-z have a higher mortality rate and are good predictors of non cause specific mortality among children under 5 and are obvious candidates for rehabilitation.

## Conclusions

Our data show MUAC to be a significant predictor of subsequent mortality and this can be used for selecting children for rehabilitation where measurement of height is not feasible. To identify the largest proportion of children who are at risk and need rehabilitation, using a combination of both MUAC and WHZ z- score for selection for rehabilitation seems appropriate through a two step screen and selection approach.

## Supporting information

S1 Table Diagnostic probability of mortality by MUAC z-scores and MUAC categories in children aged 6–23 months.pdf. (PDF)

S2 Table Diagnostic probability of mortality by MUAC z-scores and MUAC categories in children aged 24–59 months.pdf. (PDF)
