# Supplementary material for: Mid upper arm circumference as a predictor of risk of mortality in children in a low resource setting in India
Source: PLoS One. 2018 Jun 1;13(6):e0197832. doi: 10.1371/journal.pone.0197832 (PMC5983511; doi:10.1371/journal.pone.0197832)
Supplement: S1 Table — (PDF) [file pone.0197832.s001.pdf]

Table S1: Diagnostic probability of mortality by MUAC z-scores and MUAC categories in children aged 6-23 months

MUAC z-score		MUAC	
**Sensitivity/Specificity (95% CI)**			
<-3 SD	11.4 (3.2, 26.7) /98.9 (98.7, 99.1)	<115 mm	17.1 (6.6, 33.6) /98.8 (98.6, 99.0)
<-2 SD	42.9 (26.3, 60.6) / 91.8 (91.3, 92.3)	<125 mm	45.7 (28.8, 63.4) /90.2 (89.6, 90.7)
**PPV[1]/NPV[2] (95% CI)**			
<-3 SD	3.1 (0.9, 7.7) /99.7 (99.6, 99.8)	<115 mm	4.3 (1.6, 0.1) /99.7 (99.6, 99.8)
<-2 SD	1.6 (0.9, 2.6) /99.8 (99.7, 99.9)	<125 mm	1.4 (0.8, 2.3) /99.8 (99.7, 99.9)
**AUC[3] (95% CI)**			
<-3 SD	0.55 (0.50, 0.60)	<115 mm	0.58 (0.52, 0.64)
<-2 SD	0.67 (0.59, 0.76)	<125 mm	0.68 (0.60, 0.76)

[1]PPV: Positive Predictive Value

[2]NPV: Negative Predictive Value

[3]AUC: Area Under the Curve
