# Supplementary material for: Mid upper arm circumference as a predictor of risk of mortality in children in a low resource setting in India
Source: PLoS One. 2018 Jun 1;13(6):e0197832. doi: 10.1371/journal.pone.0197832 (PMC5983511; doi:10.1371/journal.pone.0197832)
Supplement: S2 Table — (PDF) [file pone.0197832.s002.pdf]

Table S2: Diagnostic probability of mortality by MUAC z-scores and MUAC categories in children aged 24-59 months

MUAC z-score		MUAC	
**Sensitivity/Specificity (95% CI)**			
<-3 SD	11.1 (2.4, 29.2) /99.2 (99.0, 99.3)	<115 mm	7.4 (0.9, 24.3) /99.9 (99.8, 99.9)
<-2 SD	25.9 (11.1, 46.3) /88.6 (88.2, 89.0)	<125 mm	11.1 (2.4, 29.2) /98.7 (98.5, 98.8)
**PPV[1]/NPV[2] (95% CI)**			
<-3 SD	1.6 (0.3, 4.5) /99.9 (99.8, 99.9)	<115 mm	6.9 (0.8, 22.8) /99.9 (99.8, 99.9)
<-2 SD	0.3 (0.1, 0.6) /99.9 (99.8, 99.9)	<125 mm	1.0 (0.2, 2.9) /99.9 (99.8, 99.9)
**AUC[3] (95% CI)**			
<-3 SD	0.55 (0.49, 0.61)	<115 mm	0.54 (0.49, 0.59)
<-2 SD	0.57 (0.49, 0.66)	<125 mm	0.55 (0.49, 0.61)

[1]PPV: Positive Predictive Value

[2]NPV: Negative Predictive Value

[3]AUC: Area Under the Curve
